# Sleep Disturbance in Mild Cognitive Impairment and Association With Cognitive Functioning. A Case-Control Study

**DOI:** 10.3389/fnagi.2018.00360

**Published:** 2018-11-09

**Authors:** Katie Palmer, Micaela Mitolo, Francesca Burgio, Francesca Meneghello, Annalena Venneri

**Affiliations:** ^1^Fondazione Ospedale San Camillo IRCCS, Venice, Italy; ^2^Policlinico S.Orsola-Malpighi, Bologna, Italy; ^3^Department of Neuroscience, University of Sheffield, Sheffield, United Kingdom

**Keywords:** sleep, cognition, MCI, cognitive impairment, aging, waking, neuropsychological testing, memory

## Abstract

**Objectives:** The aims of the current study are to (1) report the frequency of specific sleep disturbance symptoms in Mild Cognitive Impairment (MCI) and cognitive healthy older persons; (2) examine whether overall poor sleep and specific sleep disturbance symptoms are more common in persons with MCI compared to cognitive healthy older controls and; (3) examine the association between sleep disturbances and performance in general and specific cognitive domains in persons with MCI and separately in cognitive healthy older persons.

**Methods:** Data were collected at the Fondazione Ospedale San Camillo Istituto di Ricovero e Cura a Carattere Scientifico (IRCCS), Venice, Italy as part of the European VPH-DARE@IT project. We included 69 persons with MCI (mean age 75.7; *SD* = 7.7) and 72 sex-matched cognitively healthy controls (mean age 71.8; *SD* = 7.0). Participants underwent extensive neuropsychological assessment and evaluation of subjective sleep performance with the Sleep Continuity in Alzheimer’s Disease Scale(SCADS).

**Results:** A fifth of MCI patients (21.7%, *n* = 15) had poor sleep compared to 15.3% (*n* = 11) of cognitively healthy controls. MCI patients had a 3.2 higher odds of having poor sleep compared to cognitively healthy controls after adjustment for age, education, sex, and general cognitive functioning (Odds Ratio (OR) = 3.2; 95% Confidence Interval (CI) = 1.1–9.2). Persons who reported waking up twice or more during the night had higher odds of being MCI compared to those who never wake or wake only once (OR = 2.6; 95% CI = 1.1–6.1). In MCI patients, poor sleep was associated with better general cognitive functioning and short-term working memory, whereas in cognitive healthy older persons poor sleep was associated with impairment in episodic memory performance and executive functioning.

**Discussion:** Our results confirm previous studies showing that sleep disturbances are common in MCI, and this may be due to an ongoing neurodegenerative process rather than a symptom of cognitive impairment. Future research with objective sleep measurements are needed in MCI as well as interventions to improve sleep with the aim of preventing cognitive decline.

## Introduction

Sleep health covers a variety of dimensions, with many different definitions in the literature that generally cover a range of aspects including sleep satisfaction, timing, efficiency, and duration (Buysse, 2014). Sleep disorders are common in aging and increase with advancing age (Gadie et al., 2017). Research has shown that poor sleep is related to poorer general cognitive functioning as well as impairment in specific cognitive domains (Gadie et al., 2017). A meta-analysis concluded that persons with symptoms of insomnia show impairment in cognitive functioning in episodic memory, working memory and executive functioning (Fortier-Brochu et al., 2012). Interestingly, research also suggests that a cumulative index of sleep problems, rather than specific symptoms of poor sleep, is the biggest risk factor for poorer cognitive performance (Gadie et al., 2017). However, evidence is conflicting, with some studies reporting no association between sleep disturbance and cognition (Mecca et al., 2018), and some even reporting slightly better cognitive functioning in those with sleep problems (Kyle et al., 2017).

In addition to concurrent cognitive impairment, sleep quality has also been linked with subsequent cognitive decline over time (Virta et al., 2013; Blackwell et al., 2014) and specific aging disorders such as dementia. At least a quarter of Alzheimer’s disease (AD) patients exhibit severe sleep dysfunction (Moran et al., 2005). It is thought that sleep disorders start to occur in the preclinical phases of AD, as they have been found to predict incident dementia (Ju et al., 2013, 2014; Lim et al., 2013; Sterniczuk et al., 2013; Diem et al., 2016). Poor sleep quality has been found to be associated with Mild Cognitive Impairment (MCI) (Dlugaj et al., 2014), which often represents a prodromal phase of preclinical dementia. A third of MCI patients exhibit nighttime behaviors (Di Iulio et al., 2010), and specific symptoms have been objectively observed in these individuals, such as waking after sleep onset (Naismith et al., 2014). However, results are conflicting, with some studies reporting no link between sleep disturbances and cognitive decline (Mecca et al., 2018).

The overall objective of the current study is to contribute to this ongoing discussion and provide further data to examine the role of sleep disturbances in normal aging and MCI. We aimed to look in detail at different specific symptoms of sleep disturbances using the Sleep Continuity in Alzheimer’s Disease Scale (SCADS) (Manni et al., 2013), which is a validated Italian tool for the early identification and longitudinal monitoring of sleep disturbances in dementia. The specific aims of the current study were to: (1) report the frequency of specific sleep disturbance symptoms in MCI and cognitive healthy older persons; (2) examine whether overall poor sleep and specific sleep disturbance symptoms are more common in persons with MCI compared to cognitively healthy older controls and; (3) examine the association between sleep disturbances and performance in general and specific cognitive domains in persons with MCI, and separately in cognitive healthy older persons.

## Materials and Methods

### Study Population

Data were collected at the Fondazione Ospedale San Camillo Istituto di Ricovero e Cura a Carattere Scientifico (IRCCS), Lido, Venice, Italy as part of the European VPH-DARE@IT project^1^. The study was approved by the joint ethics committee of the Health Authority Venice 12 and San Camillo IRCCS (Protocol number 2014.08). All participants gave informed consent prior to participation in the study.

MCI patients were recruited from the outpatient memory service at the hospital. Eligible patients amongst consecutive referrals to this service were enrolled if they met clinical criteria for MCI (Petersen et al., 1999, 2001; Albert et al., 2011) and met the following exclusion criteria: diagnostic entities of clinical concern (brain tumor, hydrocephalous, lesions indicative of multiple sclerosis, large cysts, and excessive leukoaraiosis), chronic or acute cerebrovascular disease as main etiology, history of transient ischemic attacks, presence of uncontrolled brain seizures, peptic ulcer, cardiovascular disease, sick-sinus syndrome, neuropathy with conduction difficulties, proof of abnormal levels of folates, vitamin B12 or thyroid stimulating hormone, significant neuropsychiatric diagnoses such as Major Depression, Anxiety or Psychosis, treatment with medication for research purposes or with toxic effects to internal organs (e.g., drugs that could potentially be toxic to the liver or heart, anti-epileptic drugs, or psychoactive medications such as amphetamine or ketamine). Participants with significant disabilities or with MRI indication of a major diagnostic category of non-neurodegenerative nature, which could otherwise explain cognitive symptoms, were not considered for recruitment. Sleep apnea and presence of neuropsychiatric sleep disturbances were not used as exclusion criteria.

Healthy controls were recruited amongst carers and spouses of the MCI patients and through word of mouth amongst the residents of the island of Lido in Venice. The same relevant exclusion criteria used for patients were also applied for enrolment of controls.

We included 141 persons aged ≥52 years (range 52–89); 69 persons with MCI and 72 sex-matched cognitively healthy controls. Characteristics of the sample are reported in Table 1.

**Table 1:** Characteristics of the study population.

		Mild cognitive impairment (MCI) (*n* = 69)		Healthy controls (*n* = 72)		All (*n* = 141)	
Sex, female (*n*, %)		*n* = 42	60.9%	*n* = 42	58.3%	*n* = 84	59.6%
		Mean	*SD*	Mean	*SD*	Mean	*SD*

Age		75.7	7.7	71.8	7.0	73.7	7.6
Years of education		9.6	3.8	12.5	4.2	11.1	4.3
Mini-mental state examination (MMSE)	Range=19–30	25.6	2.9	28.6	1.4	27.1	2.7
Raven’s colored progressive matrices	Range=9–36	23.3	6.0	29.7	4.1	26.6	6.0
Phonemic fluency	Range=6 to 69	26.6	11.2	32.8	10.1	29.8	11.1
Semantic fluency	Range=6 to 72	27.1	11.1	39.6	8.5	33.5	11.6
Attention matrices	Range=17 to 60	42.6	9.2	52.0	6.3	47.4	9.2
Analogies	Range=4 to 28	15.2	4.5	21.0	3.6	18.2	5.0
Token test	Range=21 to 63	31.3	2.8	34.2	1.8	32.8	2.8
Rey figure – copy	Range=3 to 336	25.8	8.5	33.7	2.8	29.8	7.4
Rey figure – delayed	Range=0 to 326	6.2	4.9	14.7	5.6	10.5	6.8
Stroop – Time	Range = 0.5 to 119	39.2	22.6	23.3	9.4	31.1	18.9
Stroop – Error	Range=-1 to 30	6.6	8.2	0.5	1.2	3.4	6.5
Digit span forward	Range=4 to 8	5.3	0.9	6.0	1.0	5.7	1.0
Digit span backward	Range=2 to 6	3.6	0.9	4.3	0.9	4.0	1.0
Corsi block test	Range=2 to 8	4.4	0.9	5.0	1.1	4.7	1.1
Episodic memory – immediate	Range=0 to 18	6.5	3.8	10.4	3.8	8.5	4.2
Episodic memory – delayed	Range=0 to 24	6.8	5.2	14.5	4.5	10.7	6.2
Episodic memory – total	Range=0 to 42	13.9	8.8	24.9	7.6	19.5	9.9
Word pairing test	Range=0 to 20	8.0	4.3	11.5	3.7	9.8	4.3
Naming test	Range=8 to 20	16.9	3.0	19.1	1.5	18.0	2.6


### Neuropsychological Testing and MCI Diagnosis

An extensive neuropsychological battery was administered to all participants by a neuropsychologist, including Italian versions of the Mini-Mental State Examination (MMSE) (Measso et al., 1993), Raven’s colored progressive matrices (Basso et al., 1987), Phonemic/Semantic fluency tests (Novelli et al., 1986b), Digit Cancelation test (Orsini et al., 1987), Similarity subtest from the Wechsler Adult Intelligence Scale (Wechsler, 1981), Token test (De Renzi and Faglioni, 1978), Rey complex figure test (Caffarra et al., 2002a), Stroop test (Caffarra et al., 2002b), Corsi block test (Orsini et al., 1987), immediate and delayed word recall (Novelli et al., 1986a), Paired Associates (Novelli et al., 1986a), and the Confrontation Naming test (Novelli et al., 1986b). Test scores were corrected for age, education, and sex as necessary.

In addition to the neuropsychological testing, all participants underwent a clinical examination by a neurologist. MCI was diagnosed according to Petersen et al’s criteria (Petersen et al., 1999, 2001; Albert et al., 2011). Diagnostic status was reached by multidisciplinary consensus based on clinical, neuropsychological and neuroimaging evidence, see Lassila et al. (2018) for a description of the neuroimaging methods.

### Sleep Continuity in Alzheimer’s Disease Scale (SCADS)

**FIGURE 1:**
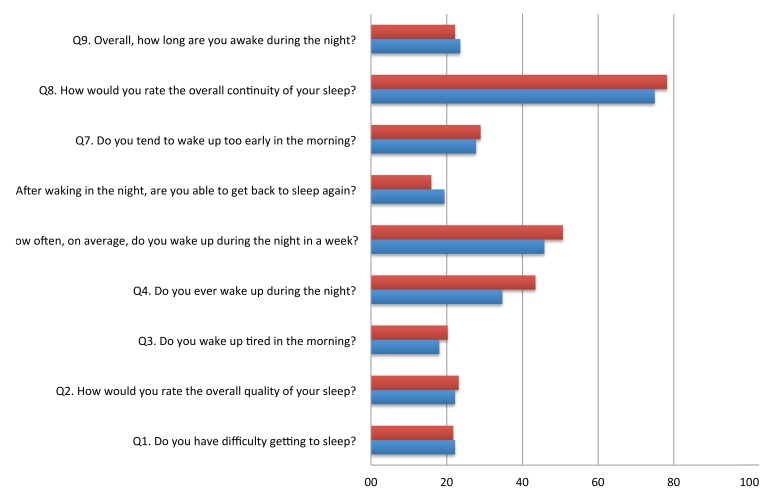
Proportion of poor scores (>2) on each item of the Sleep Continuity in Alzheimer’s Disease Scale (SCADS) sleep scale in MCI and cognitively health controls.

Sleeping behaviors were assessed during clinical evaluation. We used the SCADS scale (Manni et al., 2013, 2015), which has been validated in Italian patients with cognitive impairment. The scale includes nine self-reported questions regarding length and quality of sleep, waking during the night, and difficulties in getting to sleep; please see Figure 1 for all questions. For each question participants rated the quality of sleep according to four different responses. For the current analyses all questions were recoded so that score 1 indicates no problem and score 4 indicates very poor. Individual questions as well as the total score were investigated in the current analysis. This scale differs from the Neuropsychiatric Inventory because it assesses nine sleep-related symptom separately, thus allowing a more comprehensive insight into specific nighttime behaviors. However, it relies on self-report from the patient rather than a caregiver.

### Statistical Analysis

First, we reported sociodemographic variables (age, sex, and years of education) and mean performance on the neuropsychological tests in the whole population (*n* = 141), and separately in MCI patients (*n* = 69) and cognitively healthy controls (*n* = 72). Second, we calculated the mean scores on the 9 SCADS items. Third, SCADS item scores were dichotomized into poor (score of 3–4) vs. good/moderate (score 1–2), and we calculated the frequency of poor sleep in each of the nine items in MCI patients and cognitively healthy controls. The nine scores were then added to create an overall sleep performance score. Persons scoring one standard deviation (SD) above the mean total SCADS score were defined as having poor sleep. Multivariate logistic regression models with 95% CI were used to investigate the association between (i) overall poor sleep and MCI status and (ii) poor sleep on the individuals SCADS questions and MCI status. Finally, we stratified the sample according to MCI status to investigate the association between performance on the individual neuropsychological tests and poor sleep (total SCADS score).

## Results

The characteristics of the study population are shown in Table 1; results are shown for the whole population, and stratified according to MCI/control status. The mean age was 73.7 (*SD* = 7.6) years, with MCI patients being older than cognitively healthy controls (*t* = 3.149; *p* = 0.002). There was quite a high level of education in the sample (mean 11.1 years, *SD* = 4.3), with MCI having a lower mean number of years of education (*t* = -4.282; *p* = 0.000). As the sample was matched for sex, the proportion of females was the same in MCI and controls (60.9 vs. 58.3%). Mean scores on the MMSE and domain specific tasks of cognitive functioning are also provided in the Table.

Table 2 shows the mean score for each item of the SCADS sleep scale for MCI, controls, and the whole population. Each question ranges from 1 to 4, with a higher score indicating poorer sleep. Figure 1 illustrates the percentage of person with a poor score (3 or 4 out of 4) on each of the SCADS items, stratified according to MCI status. Overall many people rated their continuity of sleep as poor or very poor (75% of controls and 78.3% of MCI), and waking in the night was commonly reported (questions 4 and 5) in both groups. Most people reported being awake only 30 min or less during the night (question 5). Less than a quarter of people reported having regular difficulties getting to sleep (question 1).

**Table 2:** Mean scores of the Sleep Continuity in Alzheimer’s Disease Scale (SCADS) sleep scale, in persons with MCI and cognitively healthy controls.

	MCI (*n* = 69)		Cognitively healthy controls (*n* = 72)		Total population (*n* = 141)	
			
	Mean	*SD*	Mean	*SD*	Mean	*SD*
Q1. Do you have difficulty getting to sleep?	1.7	1.0	1.9	1.0	1.8	1.0
Q2. How would you rate the overall quality of your sleep?	2.8	0.6	2.9	0.7	2.9	0.6
Q3. Do you wake up tired in the morning?	1.7	1.0	1.8	0.8	1.7	0.9
Q4. Do you ever wake up during the night?	2.5	0.9	2.4	0.9	2.5	0.9
Q5. How often, on average, do you wake up during the night in a week?	2.7	1.1	2.6	1.0	2.6	1.0
Q6. After waking in the night, are you able to get back to sleep again?	1.7	0.9	1.9	1.0	1.8	1.0
Q7. Do you tend to wake up too early in the morning?	1.9	0.9	2.0	0.9	1.9	0.9
Q8. How would you rate the overall continuity of your sleep?	2.1	0.6	2.2	0.7	2.2	0.6
Q9. Overall, how long are you awake during the night?	1.7	1.0	1.8	1.1	1.7	1.0


Results of the 9 questions were summed to create a total score (max 36 points), and a dichotomous variable was created to indicate poor sleep. The mean total sleep score was 19.1. (*SD* = 4.3). Persons scoring 1 SD above the mean were classified as having poor sleep. A fifth of MCI patients (21.7%, *n* = 15) had poor sleep compared to 15.3% (*n* = 11) of cognitively healthy controls. Table 3 shows the association between poor sleep, demographic variables and MCI status. In the adjusted OR, having MCI was associated with a 3.2 higher odds of having poor sleep compared to cognitively healthy controls (adjusted OR = 3.2; 95% CI = 1.1–9.2).

**Table 3:** Logistic regression for the association between poor sleep (total SCADS score), MCI status and sociodemographic factors.

	Poor sleep (cutoff 23/36 total SCADS)			
	
	Crude		Adjusted	
		
	Odds ratio (OR)	(95% CI)	OR^∗^	(95% CI)
MCI vs. controls	1.5	(0.7–3.6)	3.2	(1.1–9.2)
Sex (female)	2.7	(1.0–7.1)	2.7	(0.9–8.2)
Age	0.98	(0.9–1.04)	0.99	(0.94–1.1)
Education	1.1	(0.9–1.0)	1.1	(0.9–1.2)
MMSE	1.2	(0.9–1.4)	1.2	(0.99–1.6)


*^∗^Adjusted for all variables in the model.*

We investigated whether having a poor score (>2) on any of the nine individual items on the SCADS scale was associated with MCI. Only one question was associated with MCI. Almost half of MCI patients woke up twice or more per night (*n* = 30, 43.5%) compared to a third of controls (*n* = 25, 24.7%). After adjusting for sex, age, education, and MMSE question 4 (Do you ever wake up during the night?) was associated with MCI status; persons who reported waking up twice or more during the night had a 2.6 higher odds of being MCI compared to those who reported never waking or waking only once (adjusted OR = 2.6; 95% CI = 1.1–6.1).

Finally, we examined whether there was an association between the total sleep score and performance on neuropsychological tests (Table 4). Logistic regression models adjusted for age, sex, and education were run separately in MCI patients and cognitively healthy controls. In cognitively healthy persons, poor sleep was significantly associated with lower performance on the Stroop test (Stroop Time OR = 1.12; 95% CI = 1.04–1.26) and episodic memory total score (OR = 0.89; 95% CI = 0.79–1.00), and there was a borderline significance for episodic memory immediate (*p* = 0.093) and delayed recall (*p* = 0.054). In contrast, in MCI patients, poor sleep scores were associated with better cognitive performance on the MMSE (OR = 1.42; 95% CI = 1.03–1.94) and the Corsi block test (OR = 2.98; 95% CI = 1.14–7.76).

**Table 4:** Association between total sleep score (higher score indicates worse sleep) with neuropsychological tests, stratified by MCI/control status.

	Healthy controls (*n* = 72)		MCI (*n* = 69)	
		
	OR^∗^	*(95%CI)*	OR^∗^	*(95%CI)*
MMSE	0.79	0.51–1.21	1.42	1.03–1.94
Raven’s progressive matrices	0.97	0.81–1.16	1.02	0.90–1.16
Phonemic fluency	0.98	0.92–1.06	1.02	0.96–1.09
Semantic fluency	0.96	0.87–1.06	1.01	0.95–1.07
Attention matrices	1.01	0.89–1.13	1.02	0.95–1.10
Analogies	0.56	1.07–1.31	1.04	0.89–1.22
Token test	1.82	0.94–3.53	1.33	0.93–1.91
Rey figure – copy	0.89	0.70–1.13	1.07	0.98–1.17
Rey figure – delayed	0.88	0.76–1.01	1.04	0.91–1.19
Stroop – Time	1.14	1.04–1.26	0.98	0.94–1.01
Stroop – Error	1.04	0.62–1.73	0.94	0.85–1.04
Digit span forward	0.92	0.43–1.95	1.09	0.48–2.44
Digit span backward	0.91	0.43–1.96	0.79	0.40–1.56
Corsi block test	0.92	0.48–1.74	2.98	1.14–7.76
Episodic memory – immediate	0.82	0.66–1.03	1.06	0.89–1.26
Episodic memory *–* delayed	0.84	0.69–1.03	1.11	0.98–1.27
Episodic memory *–* total	0.89	0.79–1.00	1.03	0.96–1.10
Word pairing test	0.94	0.78–1.14	1.12	0.96–1.30
Naming test	1.07	0.67–1.72	1.13	0.87–1.47


*^∗^Adjusted OR calculated with logistic regression, with adjustment for age, sex, and education.*

## Discussion

Our study demonstrated that persons with MCI are more than three times more likely to have overall poor sleep than cognitive healthy controls, even after adjustment for general cognitive functioning. The specific symptom of waking during the night is more common in MCI patients than controls. We also found that overall self-reported sleep performance is differentially related to cognitive functioning in MCI and controls. In MCI patients, persons with better general cognitive performance (MMSE) and short-term working memory (Corsi block test) have poorer sleep scores. In contrast, sleep disturbances are associated with poorer episodic memory performance and executive functioning in cognitive healthy controls.

Our results are in line with previous studies reporting an association between poor sleep quality and MCI (Dlugaj et al., 2014). Interestingly, the only symptom we found to specifically be related to MCI was waking during the night, which supports previous objective reports that sleep fragmentation occurs in the prodromal phases of AD, predicting subsequent dementia (Lim et al., 2013). Further, results from a sleep laboratory in which patients underwent overnight assessment with dim light melatonin onset assessment showed that MCI patients had greater wake after sleep onset, which was likely due to alterations in the timing of melatonin secretion onset and amount (Naismith et al., 2014). These results back the hypothesis that sleep disorders are related to AD and already begin in the prodromal phase of the disease (Ju et al., 2013, 2014; Lim et al., 2013; Sterniczuk et al., 2013).

Much recent evidence has focused on establishing the biological mechanisms through which sleep problems are related to neurodegeneration and cognitive decline. One explanation may be due to degeneration at the subcortical level. Degeneration of the hypothalamus occurs in AD (Iacono and Sandyk, 1987; Vercruysse et al., 2018), and this area is important for sleep regulation (Ono and Yamanaka, 2017). There is also evidence of a link between sleep disturbance and subcortical infarcts (Lim et al., 2016). Sleep disturbances in AD are also influenced by tau AD pathology (Holth et al., 2017), and there is also some suggestion that hypothalamus dysfunction may be due to reduced acetylcholine (Iacono and Sandyk, 1987).

There is a link between sleep disturbances and beta-amyloid deposition as measured by PET (Spira et al., 2013) and it has been proposed that the relationship may be bi-directional with poor sleep contributing to amyloid deposition but also sleep being disrupted as a result of amyloid plaque formation (Ju et al., 2014). Mice studies have shown that neurotoxic waste products such as beta-amyloid are cleared during sleep (Xie et al., 2013). It has also been suggested that sleep and cognitive decline may be related through stress and hormonal dysregulation (Maggio et al., 2013). Further, circadian rhythms contribute to memory formation possibly through molecular oscillator pathways such as the cAMP signaling cascade, see Gerstner and Yin (2010) for a review.

Our results showed that persons with MCI are more than three times more likely to have sleep problems than cognitively healthy controls even after adjustment for MMSE. This suggests that the sleeping problems are not related to a drop in cognitive functioning *per se*, but rather the sleep problems may reflect an ongoing neurodegenerative process, the mechanisms of which are discussed above.

Interestingly, in our sample, poor sleep was associated with better cognitive functioning in the MCI patients, in terms of general cognitive functioning and short term working memory. These results are somewhat surprising; although there are previous studies that have reported that persons with sleep problems such as insomnia have slightly better cognitive functioning (Kyle et al., 2017). There are two potential explanations for these results. First, as the SCADS is a self-rated scale, it is possible that MCI patients with a higher MMSE score are in a milder phase of the syndrome and are able to better report their sleep performance with more accuracy. Second, these milder MCI cases may be more aware of their prognosis and experience stress and worry about an impending dementia, which consequently affects their sleep. Sleep quality is strongly associated with both anxiety and depression (Gadie et al., 2017), which are also common symptoms in MCI (Di Iulio et al., 2010; Palmer et al., 2010). It is also worth noting that MCI is a heterogeneous syndrome, which does not always progress to AD and, thus, there may be other underlying etiologies that cause the cognitive impairment but do not cause sleep disturbances.

Our study also found that in cognitively healthy participants poor sleep was related to worse performance in episodic memory and executive functioning, in agreement with previous findings (Wilckens et al., 2014; Bernstein et al., 2018); low sleep efficiency can lead to loss of attention, resulting in more errors on executive tasks like the Stroop test. It has also been hypothesized that stress hormones have a role in sleep disruption and cognition during aging (Maggio et al., 2013). Another hypothesis is that poor sleep is related to impaired cognitive functioning via a third pathway, such as neuropsychiatric symptoms. Studies suggest that night-time behaviors often cluster with other affective symptoms, particularly depression and anxiety, and that up to two-thirds of non-demented persons with affective syndromes characterized by depression, anxiety and nighttime behaviors take psychotropic medications (Cravello et al., 2011), which may also affect cognitive functioning. Unfortunately, in the current study we were not able to investigate the potential confounding effect of prescription drugs or neuropsychiatric symptoms.

There are several strengths of our study. Both cases and controls underwent the same rigorous evaluation for MCI diagnosis and extensive neuropsychological assessment. We also used a sleep scale that has been validated in an Italian population and is designed to address sleep problems related to cognitive decline and dementia. However, several limitations should also be discussed. First, as the study is cross-sectional we cannot determine any causal relationships between cognitive functioning and sleep disturbances. Second, the sleep scale was self-reported and we cannot exclude that a reporting bias may have occurred due to cognitive impairment in the MCI patients. Further, although difficult to measure in a large sample, an objective measure of sleep performance would provide more accurate results. Third, although cases and controls were matched by sex, and we made adjustments for sociodemographics, there may be other unmeasured confounding that may affect the relationships investigated, particularly psychiatric factors such as depression and anxiety or medication use. Another important limitation is that we were not able to establish whether waking during the night was due to obstructive sleep apnea, which is known to cause cognitive deficits and increase the risk of MCI and dementia (Gagnon et al., 2014). Finally, although statistically significant, the reported OR on the association between MCI and poor sleep should be interpreted with caution due to the lower CI being close to 1.

Our results support previous findings in the literature that suggest that sleep problems are common in MCI, which may be relevant for clinicians to consider during clinical evaluation. There is much discussion about whether specific interventions to improve sleep will help to prevent AD (Spira and Gottesman, 2017), but as there is no evidence that interventions will have a successful preventative effect and may be difficult to implement on a large scale, there are currently no guidelines regarding this issue. Future research is needed, with specially designed clinical trials on pharmaceutical and non-pharmaceutical interventions focused on different outcomes, including prevention of AD but also cognitive impairment in general in older adults. Considering the potential side effects of pharmaceutical drugs, lifestyle interventions such as exercise and other non-pharmaceutical interventions may be of particular interest. A recent review concluded that there is insufficient evidence on the role of cognitive behavioral therapy for insomnia for improving cognitive performance (Herbert et al., 2018). One issue in the current literature is the wide range of sleep disorders and symptoms and variation in defining the problems, from insomnia, sleep-disordered breathing, and inefficient sleeping, to delayed sleeping. Objective measures of sleep performance such as actigraphy, bed sensors, and eyelid movement sensors (Van de Water et al., 2011) are needed on a wider scale in MCI patients to better understand the role of sleeping in this syndrome.

## Conclusion

Our results show that sleep disturbances are impaired in MCI, possibly due to ongoing neurodegenerative processes, and that in cognitive healthy controls poor sleep is associated with poor episodic memory and executive functioning.

## Author Contributions

FB, MM, FM, and AV collected data and contributed to critical revision of the manuscript. KP, MM, FM, and AV contributed to hypothesis and study design. KP analyzed the data and wrote the manuscript. KP, MM, and AV interpreted the result.

## Conflict of Interest Statement

The authors declare that the research was conducted in the absence of any commercial or financial relationships that could be construed as a potential conflict of interest.

## Footnotes

1 www.vph-dare.eu
